# Editorial: Pushing the boundaries in acute ischemic stroke treatment

**DOI:** 10.3389/fneur.2023.1245890

**Published:** 2023-08-14

**Authors:** Bastian Volbers, Bernd Kallmünzer, David J. Seiffge

**Affiliations:** ^1^Department of Neurology, Inselspital, Bern University Hospital, University of Bern, Bern, Switzerland; ^2^Department of Neurology, University of Erlangen-Nürnberg, Erlangen, Germany

**Keywords:** acute ischemic stroke, thrombolysis (for acute ischemic stroke), mechanical thrombectomy, extended time window, telestroke network, non-contrast computer tomography (NCCT), cardiac myxoma, intracerebral hemorrhage (ICH)

Over the past few decades, there have been significant advancements in the treatment of acute ischemic stroke. Intravenous thrombolysis and mechanical thrombectomy have become established procedures, and the time window for treatment has been extended. However, it is important to note that still only a small percentage, ~10–20%, of all ischemic stroke patients are eligible for acute reperfusion treatment. This situation gives rise to various challenges. Patient selection and treatment selection remain complex tasks, as many patients arrive at the hospital too late for intervention, the availability of multimodal neuroimaging may be limited, and adverse events can hinder the effectiveness of treatment.

Concerning the limited availability of multimodal imaging to select ischemic stroke patients for intravenous thrombolysis (IVT) in the extended time window, Emde et al. performed a review about existing data to support IVT treatment based on non-contrast CT (NCCT). This is of interest, as multimodal imaging may not be widely available in rural areas as well as in low- or middle-income countries and primary stroke centers (1). Mainly based on the TRUST-CT registry (2) and the TWIST trial (using tenecteplase) (3) current data suggests that, particularly for patients with wake-up strokes and those presenting within 6 h of symptom onset, IVT is safe and could lead to favorable functional outcomes (2). However, favorable outcomes could not be shown if tenecteplase is used (3). In cases where there is a high Alberta Stroke Program Early CT Score (ASPECTS) and a clinical mismatch between symptom severity and radiological findings, treatment may be considered, even in the absence of advanced imaging. However, since randomized controlled data is scarce, thrombolysis should be evaluated with care especially if tenecteplase is used, ASPECTS is low and time since onset comes close to or exceeds 6 h. Regarding mechanical thrombectomy in the late time window, treatment decisions based on NCCT imaging (ASPECTS>5) also appear to be safe and effective (4), and this is more commonly used in clinical practice when advanced imaging is not available (1).

However, if there is an indication to perform mechanical thrombectomy, a highly specialized infrastructure is needed to get the patient to the treatment (-site) in time. Here, Riegler et al. examined the time-to-care metrics in an established tele-stroke-network in northern Germany. Their study presents the findings from an analysis of 92 stroke patients eligible for mechanical thrombectomy (MT) who initially received treatment at a hospital without MT capabilities and were subsequently transferred to a comprehensive stroke center (CSC). The authors compared patients initially admitted to rural hospitals within a telestroke network with those presenting at metropolitan primary stroke centers. They found that the administration of intravenous thrombolysis (IVT) was significantly faster in metropolitan hospitals (37 vs. 65 min, *p* < 0.01). This is not surprising, since metropolitan hospitals do not require additional tele-video consultation before thrombolysis. However, the delay from arrival at the CSC to groin puncture was shorter for patients transferred from rural telestroke centers (42 vs. 60 min, *p* < 0.01), while the overall treatment time from arrival at the primary hospital to groin puncture did not significantly differ between the two groups (210 vs. 208 min, *p* = 0.96). These results again demonstrate the importance and effectiveness of telestroke networks in treating stroke patients in rural areas, especially if a transfer to a stroke center is needed. However, it requires optimal organization, clear and rigorous standard operating procedures (SOPs), strict quality monitoring, and regular, high quality training to ensure high process quality.

Further publications of our Research Topic dealt with areas that have been less well elucidated so far including safety and efficacy of mechanical thrombectomy (MT) in large vessel occlusions (LVO) associated with cardiac myxoma and the natural history of carotid-artery free-floating thrombus. In their case series including eleven patients with left-atrium cardiac myxoma associated LVO treated with MT, Rao et al. found a favorable outcome rate of 60% at 3 months. Interestingly, they performed a histopathological examination of the retrieved thrombus material of five patients and found myxoma components within the thrombus. In this context, it is reassuring and encouraging that, even if thrombus formation may differ from “classical” embolic thrombi, mechanical thrombectomy is an effective and safe treatment option in those patients. Müller et al. found a high recurrence rate of ischemic stroke (20%) in patients with carotid-artery free-floating thrombus within the first week after the index event. While those results confirm the general clinical experience, the optimal management of those patients urgently needs to be elucidated.

Altenbernd et al. assessed the sensitivity and specificity of CT-angiography (CTA) compared to digital subtraction angiography (DSA) to further push the diagnostic boundaries in patients with intracerebral hemorrhage (ICH). Although DSA remains the preferred diagnostic modality for detecting macrovascular causes of ICH, CTA can effectively investigate most cases of lobar ICH and identify the underlying cause of bleeding. The authors observed false-negative findings in CTA only in 3/125 patients (2%; two arteriovenous malformations (AVMs) measuring < 2 cm and one small dural fistula). Thus, further research may focus on the selection of those patients with negative CTA results who may benefit from an additional DSA.

Finally, we included a study protocol by Staszewski et al. for a prospective, open label, single-center study assessing the efficacy of Cerebrolysin treatment as an add-on therapy to mechanical thrombectomy. Cerebrolysin is associated with cytoprotective effects by promoting cellular survival while inhibiting glutamate excitotoxicity, free radical formation, and pro-inflammatory mediators such as TNF-α, IL-1β and IL-6. The authors represent the hypothesis that the addition of Cerebrolysin within 8 h of stroke onset, in carefully selected patients based on clinical and radiological criteria (moderate to severe stroke, small ischemic core and good collateral status), can enhance the effectiveness of thrombectomy. Suggested mechanisms include the initiation of cytoprotective mechanisms and the prevention of reperfusion injury.

We congratulate all contributors for their outstanding and forward-looking work and hope that our Research Topic will contribute to improving the treatment and care of patients with ischemic stroke or intracerebral hemorrhage.

## Author contributions

BV, BK, and DS made substantial contributions to the conception or design of the work, provide approval for publication of the content, and agreed to be accountable for all aspects of the work in ensuring that questions related to the accuracy or integrity of any part of the work are appropriately investigated and resolved. BV drafted the manuscript. BK and DS revised it critically for important intellectual content. All authors contributed to the article and approved the submitted version.
